# Clinical Outcomes and Hospital Utilization Among Patients Undergoing Bariatric Surgery With Telemedicine Preoperative Care

**DOI:** 10.1001/jamanetworkopen.2022.55994

**Published:** 2023-02-10

**Authors:** Callie Hlavin, Phoebe Ingraham, Tamara Byrd, Nathan Hyre, Lucine Gabriel, Nishant Agrawal, Laura Allen, Tanya Kenkre, Andrew Watson, Murat Kaynar, Bestoun Ahmed, Anita Courcoulas

**Affiliations:** 1 Department of Surgery, University of Pittsburgh, Pittsburgh, Pennsylvania; 2 Department of Anesthesiology and Perioperative Medicine, University of Pittsburgh, Pittsburgh, Pennsylvania; 3 School of Medicine, University of Pittsburgh, Pittsburgh, Pennsylvania; 4 Epidemiology Data Center, University of Pittsburgh, Pittsburgh, Pennsylvania

## Abstract

**Importance:**

Bariatric surgery is the mainstay of treatment for medically refractory obesity; however, it is underutilized. Telemedicine affords patient cost and time savings and may increase availability and accessibility of bariatric surgery.

**Objective:**

To determine clinical outcomes and postoperative hospital utilization for patients undergoing bariatric surgery who receive fully remote vs in-person preoperative care.

**Design, Setting, and Participants:**

This cohort study comparing postoperative clinical outcomes and hospital utilization after telemedicine or in-person preoperative surgical evaluation included patients treated at a US academic hospital. Participants underwent laparoscopic Roux-en-Y gastric bypass or laparoscopic sleeve gastrectomy after telemedicine or in-person preoperative surgical evaluation between July 1, 2020, to December 22, 2021, or January 1, 2018, to December 31, 2019, respectively. Follow-up was 60 days from date of surgery.

**Exposures:**

Telemedicine-based preoperative care.

**Main Outcomes and Measures:**

Clinical outcomes, including operating room delay, procedure duration, length of hospital stay (LOS), and major adverse events (MAE), and postoperative hospital resource utilization, including emergency department (ED) visit or hospital readmission within 30 days of the surgical procedure.

**Results:**

A total of 1182 patients were included; patients in the telemedicine group were younger (mean [SD] age, 40.8 [12.5] years vs 43.0 [12.2] years; *P* = .01) and more likely to be female (230 of 257 [89.5%] vs 766 of 925 [82.8%]; *P* = .01) compared with the control group. The control group had a higher frequency of comorbidity (887 of 925 [95.9%] vs 208 of 257 [80.9%]; *P* < .001). The telemedicine group was found to be noninferior to the control group with respect to operating room delay (mean [SD] minutes, 7.8 [25.1]; 95% CI, 5.1-10.5 vs 4.2 [11.1]; 95% CI, 1.0-7.4; *P* = .002), procedure duration (mean [SD] minutes, 134.4 [52.8]; 95% CI, 130.9-137.8 vs 105.3 [41.5]; 95% CI, 100.2-110.4; *P* < .001), LOS (mean [SD] days, 1.9 [1.1]; 95% CI, 1.8-1.9 vs 2.1 [1.0]; 95% CI, 1.9-2.2; *P* < .001), MAE within 30 days (3.8%; 95% CI, 3.0%-5.7% vs 1.6%; 95% CI, 0.4%-3.9%; *P* = .001), MAE between 31 and 60 days (2.2%; 95% CI, 1.3%-3.3% vs 1.6%; 95% CI, 0.4%-3.9%; *P* < .001), frequency of ER visits (18.8%; 95% CI, 16.3%-21.4% vs 17.9%; 95% CI, 13.2%-22.6%; *P* = .03), and hospital readmission (10.1%; 95% CI, 8.1%-12.0% vs 6.6%; 95% CI, 3.9%-10.4%; *P* = .02).

**Conclusions and Relevance:**

In this cohort study, clinical outcomes in the telemedicine group were not inferior to the control group. This observation suggests that telemedicine can be used safely and effectively for bariatric surgical preoperative care.

## Key Points

**Question:**

Can fully remote patient evaluation be utilized in bariatric surgery with equivalent patient outcomes and hospital utilization as compared with in-person patient evaluation?

**Findings:**

In this cohort study of 1182 patients treated at a US academic hospital, clinical outcomes and postoperative hospital utilization in the telemedicine group were found to be noninferior to those in the in-person group.

**Meaning:**

These results suggest that telemedicine may provide more accessibility and flexibility to patients receiving preoperative care for bariatric surgery.

## Teaser

This cohort study conducted at a US academic hospital compares clinical outcomes and follow-up hospitalization of patients receiving bariatric surgery utilizing telemedicine for preoperative care with an in-person control group.

## Introduction

Telemedicine (or telehealth) has emerged over the past several decades as a practical and beneficial health care adjunct. It has the potential to revolutionize the way health care is provided, offering improved efficiency, better flexibility, and time and cost savings to patients, clinicians, and hospital centers.^1,2^

In the field of surgery, telemedicine is utilized by a broad range of subspecialties and has demonstrated feasibility, diagnostic accuracy, high levels of patient and clinician satisfaction, and acceptable patient outcomes.^3,4,5,6,7,8,9^ Additionally, day-of-surgery cancellation rates are unchanged.^10,11^ We define *total preoperative surgical telemedicine* as the patient completing all preoperative surgical clinical encounters virtually. In this definition, the patient meets the surgeon in-person for the first time on the day of surgery.

The prevalence of obesity is steadily increasing in the US across race, sex, and age and disproportionately affects patients in minority racial and ethnic groups and those of low socioeconomic status. Telemedicine may provide an opportunity for enhanced efficiency and equitability in access to bariatric surgical care.^12^ Previous studies have shown that telemedicine is feasible in the bariatric population for preoperative education and postoperative monitoring.^13,14^ Preoperative teleconferencing between care teams can also reduce travel distance, time, and costs for patients.^15,16,17^ However, there are no current studies investigating bariatric surgery patient outcomes after exclusive use of telemedicine for preoperative surgical evaluation with the first face-to-face encounter between patient and surgeon occurring on the day of surgery. The objective of this study is to measure patient-centered outcomes after total telemedicine preoperative bariatric surgical care. We hypothesized that the telemedicine group would have noninferior clinical outcomes and postoperative hospital utilization compared with the control, in-person group.

## Methods

We conducted a single-institution, retrospective, noninferiority cohort study in accordance with Strengthening the Reporting of Observational Studies in Epidemiology (STROBE) reporting guidelines. The University of Pittsburgh Medical Center Quality Review Committee reviewed and approved this study, and therefore it did not require additional institutional review board oversight. Informed consent of study participants was waived because it was deemed to present no more than minimal risk of harm to patients.

We used patient data collected from a contemporary cohort of patients who underwent laparoscopic Roux-en-Y gastric bypass (RNY) or sleeve gastrectomy (SG) between July 1, 2020, and December 22, 2021, after total telemedicine preoperative surgical evaluation. Telemedicine patients were identified through a query of surgical appointment records to identify patients that had preoperative surgical evaluation exclusively through telemedicine. Telemedicine visits were video-based and were performed by a combination of surgical attending physicians, fellows, residents, advance practice clinicians, and nurses. The telemedicine cohort was compared with a historical control cohort of patients who underwent RNY or SG between January 1, 2018, and December 31, 2019, after traditional in-person surgical evaluation. The control cohort was identified through operative records. Telemedicine patients were gathered from a single-surgeon census. The control cohort was gathered from multiple bariatric surgeons at a single institution.

Patient demographic, socioeconomic, and medical variables were collected by research assistants through medical record review. Demographic variables included age, sex, race, ethnicity, and primary language. Race and ethnicity were included because prior studies suggest longstanding inequalities in the treatment of racial and ethnic minorities may affect surgical outcomes. Socioeconomic variables included marital status, employment status, and insurance coverage. Medical variables collected were preoperative body mass index (BMI; calculated as weight in kilograms divided by height in meters squared), recent postoperative BMI, and presence of selected comorbidities, including anxiety or depression, type 2 diabetes, gastroesophageal reflux disease, hypertension, chronic kidney failure, nonalcoholic fatty liver disease, obstructive sleep apnea, lower extremity osteoarthritis, polycystic ovarian syndrome, pulmonary embolism and deep vein thrombosis, current smoking, and current substance abuse.

### Statistical Analysis

Patient factors were compared by telemedicine status using χ^2^ tests for categorical variables and *t* tests for continuous measures. *P* values ≤.05 signified statistically significant differences between the telemedicine and control groups.

Clinical outcomes included operating room delay or time to operating room (measured in minutes), procedure duration (minutes), length of stay (days), and major adverse events (MAE) within the first 30 days and between 31 to 60 days postoperatively. MAE were defined as death, reoperation, percutaneous or endoscopic intervention, pulmonary embolism and deep vein thrombosis, and failure to be discharged from the hospital within 30 days of the operation. Operating room delay was measured as the difference between the documented time that the patient entered the operating room and the scheduled procedure start time. This variable was measured for procedures scheduled as the first case of the operating day. Hospital utilization variables collected were emergency department (ED) visit or readmission to the hospital within 30 days of the procedure.

Continuous outcomes included time to operating room (in minutes), procedure duration (minutes), and length of stay (LOS) (days) with margins of inferiority set to 10 minutes, 45 minutes, and 1 day, respectively. Margins were based on clinical outcome and generally approximate a 50% change in the standard of care. The accepted time between surgical cases is 20 to 30 minutes. A delay of 10 minutes (33% to 50% change) could affect the surgeon’s operating schedule for the remainder of the day. Bariatric surgery procedure duration typically ranges from 90 to 180 minutes with longer times for RNY compared with SG. We selected 45 minutes (50% change) as the margin of inferiority. The accepted LOS after bariatric surgery is 2 days^18^; we deduced that a change of 1 day (50% change) would signal a significant change. The Farrington-Manning score test for noninferiority was used to compare categorical outcomes with the margin for noninferiority set to 0.7. Missing data were excluded in analyses. For this analysis, the null hypothesis states that the telemedicine group is inferior to the control group. *P* values <.05 reject the null hypothesis and signify that outcomes in the telemedicine group were not inferior to the control group.

We conducted stepwise logistic regression modeling to estimate each of 4 outcomes: 30-day ER admission; 30-day Hospital readmission; 30-day MAE; and 60-day MAE. In step 1, univariate models estimating the effect of telemedicine status on each outcome were evaluated. In intermediate steps, we evaluated the effects of additional explanatory variables on each outcome while retaining telemedicine status as a covariate as it is central to our research question. The additional variables we considered were age, employment status, type of insurance, presence of comorbidities, and type of bariatric procedure. Variables that demonstrated a *P* value ≤.20 were included in multivariate models projecting each outcome. Combinations of variables were evaluated for stability of parameter estimates. In the final step, the explanatory variables from the intermediate steps that demonstrated a *P* value ≤.05 were retained in the final model for each outcome. SAS version 9.4 (SAS Institute Inc) was used for analyses.

## Results

### Demographic, Socioeconomic, and Medical Variables

Patients in the telemedicine group (257 patients) were younger (mean [SD] age, 40.8 [12.5] years vs 43.0 [12.2]; *P* = .01) and more likely to be female (230 of 257 [89.5%] vs 766 of 925 [82.8%]; *P* = .01) compared with the control group (925 patients) (Table 1). Race and ethnicity between the 2 groups were not statistically different (telemedicine group: 53 [20.6%] Black, 0 Hispanic or Latinx, 192 [74.7%] White vs control group: 185 [20.0%] Black, 10 [1.1%] Hispanic, 711 [76.9%] White; *P* = .20). In the control group, there was 1 participant with a missing race variable. Control and telemedicine groups differed significantly in primary language. English was preferred in a higher percentage of the control cases compared with the telemedicine group (910 of 925 [98.4%] vs 255 of 257 [99.2%]).

**Table 1.** Patient Factors by Telemedicine Status

Variable	Cases, No. (%)		*P* value
	Control (n = 925)	Telemedicine (n = 257)	
**Demographic**			
Age, mean (SD), y	43.0 (12.2)	40.8 (12.5)	.01
Sex			
Female	766 (82.8)	230 (89.5)	.01
Male			
Race^a^			
Black	185 (20.0)	53 (20.6)	.20
Multiple	4 (0.4)	0	
Unknown	18 (1.9)	11 (4.3)	
White	711 (76.9)	192 (74.7)	
Other	6 (0.6)	1 (0.4)	
Ethnicity			
Hispanic/Latinx	10 (1.1)	0	.12
Not Hispanic	854 (92.3)	243 (94.6)	
Unknown	61 (6.6)	14 (5.4)	
Primary language			
English	910 (98.4)	255 (99.2)	.02
Spanish	1 (0.1)	2 (0.8)	
Other/unknown	14 (1.5)	0	
Marital status			
Single	367 (39.7)	123 (47.9)	.06
Married	411 (44.4)	102 (39.7)	
Separated	14 (1.5)	1 (0.4)	
Divorced	93 (10.1)	22 (8.6)	
Widowed	19 (2.1)	1 (0.4)	
Unknown	21 (2.3)	38 (3.1)	
Employment			
Employed	601 (65.0)	158 (61.5)	<.001
Unemployed	136 (14.7)	60 (23.4)	
Retired	46 (5.0)	3 (1.2)	
Student	20 (2.2)	11 (4.3)	
Disabled	37 (4.0)	0	
Homemaker	0	0	
Unknown	85 (9.2)	25 (9.7)	
Insurance			
Private	592 (64.0)	244 (94.9)	<.001
Medicare	156 (16.9)	5 (2.0)	
Medicaid	63 (6.8)	1 (0.4)	
Uninsured	85 (9.2)	7 (2.7)	
Unknown	29 (3.1)	0	
**Medical**			
BMI, mean (SD)			
Preoperative measurement	47.2 (7.8)	47.4 (7.5)	.72
Recent measurement^b^	34.2 (7.7) [805]	36.2 (7.9)	<.001
Comorbidities	887 (95.9)	208 (80.9)	<.001
Anxiety or depression	499 (54.0)	108 (42.0)	.001
Type 2 diabetes	175 (18.9)	22 (8.6)	<.001
Dyslipidemia	291 (31.5)	52 (20.2)	.001
Gastroesophageal reflux disease	430 (46.5)	80 (31.1)	<.001
Hypertension	448 (48.4)	90 (35.0)	<.001
Chronic kidney disease	23 (2.5)	3 (1.2)	.24
Nonalcoholic fatty liver disease	110 (11.9)	22 (8.6)	.15
Obstructive sleep apnea	648 (70.1)	73 (28.5)	<.001
Lower extremity osteoarthritis	82 (8.9)	14 (5.4)	.09
Polycystic ovarian syndrome	111 (12.0)	28 (10.9)	.66
Pulmonary embolism and deep vein thrombosis	63 (6.8)	13 (5.1)	.39
Current smoker	25 (2.7)	12 (4.7)	.15
Current substance abuse	8 (0.9)	1 (0.4)	.69
Procedure			
Laparoscopic Roux-En-Y	571 (61.7)	137 (53.3)	.01
Laparoscopic sleeve gastrectomy	354 (38.3)	120 (46.7)	

^a^ Data on race were missing for 1 patient in the control group—percentages are out of 924 patients. Details of the other category for race were not provided in the data set.

^b^ Total 805 patients.

Marital status was not statistically different between the telemedicine and control groups. Employment status differed, with a higher proportion of patients employed in the control group compared with the telemedicine group (601 of 925 [65.0%] vs 158 of 257 [61.5%]). Among the control group, as compared with the telemedicine group, there were more retirees (46 of 925 [5.0%] vs 3 of 257 [1.2%]) and disabled persons (37 of 925 [4.0%] vs none), and fewer unemployed patients (136 of 925 [14.7%] vs 60 of 257 [23.4%]) and students (20 of 925 [2.2%] vs 11 of 257 [4.3%]) (*P* < .001). Insurance status also differed significantly. The control group had more Medicare enrollees (156 of 925 [16.9%] vs 5 of 257 [2.0%]) and Medicaid enrollees (63 of 925 [6.8%] vs 1 of 257 [0.4%]) as well as uninsured patients (85 of 925 [9.2%] vs 7 of 257 [2.7%]) and those with unknown insurance status (29 of 925 [3.1%] vs none). Among the telemedicine group, a higher proportion of patients had private insurance coverage compared with the control group (244 of 257 [94.9%] vs 592 of 925 [64.0%]; *P* < .001) (Table 1).

Preoperative body mass index (BMI; calculated as weight in kilograms divided by height in meters squared) was not different between the telemedicine and control groups. The most recent postoperative BMI in the medical record was lower in the control group compared with the telemedicine group (mean [SD] BMI, 34.2 [7.7] vs 36.2 [7.9]; *P* < .001). Recent postoperative BMI was included in only those patients who had a BMI measurement within 2 years of data collection. For the control group, 805 of 925 patients had an eligible recent BMI measurement. The presence of any comorbidity differed significantly between the control and telemedicine groups (887 of 925 [95.9%] vs 208 of 257 [80.9%]; *P* < .001). Patients in the control group had significantly higher frequency of anxiety or depression (499 of 925 [54.0%] vs 108 of 257 [42.0%]; *P* < .001), type 2 diabetes (175 of 925 [18.9%] vs 22 of 257 [8.6%]; *P* < .001), dyslipidemia (291 of 925 [31.5%] vs 52 of 257 [20.2%]; *P* = .001), gastroesophageal reflux disease (430 of 925 [46.5%] vs 80 of 257 [31.1%]; *P* < .001), hypertension (448 of 925 [48.4%] vs 90 of 257 [35.0%]; *P* < .001), and obstructive sleep apnea (648 of 925 [70.1%] vs 73 of 257 [28.5%]; *P* < .001). There was no difference in frequency of chronic kidney disease, nonalcoholic fatty liver disease, lower extremity osteoarthritis, polycystic ovarian syndrome, pulmonary embolism and deep vein thrombosis, and current smoking or current substance abuse between the groups (Table 1).

We observed significant differences in the frequency of laparoscopic RNY gastric bypass and laparoscopic SG between the telemedicine and control groups (*P* = .01). A higher proportion of patients in the control group underwent RNY (571 of 925 [61.7%] vs 137 of 257 [53.3%]) compared with SG (354 of 925 [38.3%] vs 120 of 257 [46.7%]) (Table 1).

### Clinical Outcomes and Hospital Resource Utilization

We observed that clinical outcomes in the telemedicine group were not inferior to those in the control group. There was no difference between the control and telemedicine groups with regards to operating room delay (time to operating room) (margin for inferiority set to 10 minutes with mean [SD] minutes, 7.8 [25.1]; 95% CI, 5.1-10.5 vs 4.2 [11.1]; 95% CI, 1.0-7.4; *P* = .002), procedure duration (margin of inferiority set to 45 minutes with mean [SD] minutes, 134.4 [52.8]; 95% CI, 130.9-137.8 vs 105.3 [41.5]; 95% CI, 100.2-110.4; *P* < .001), length of hospital stay (margin of inferiority set to 1 day with mean [SD] days, 1.9 [1.1]; 95% CI, 1.8-1.9 vs 2.1 [1.0]; 95% CI, 1.9-2.2; *P* < .001), major adverse events within 30 days (3.8%; 95% CI, 3.0% - 5.7% vs 1.6%; 95% CI, 0.4%-3.9%; *P* = .001), and MAEs between 31 and 60 days (2.2%; 95% CI, 1.3%-3.3% vs 1.6%; 95% CI, 0.4%-3.9%; *P* < .001) (Table 2).

**Table 2.** Clinical Outcomes and Hospital Utilization by Telemedicine Status

Variable	Cases, No. (%) [95% CI]		*P* value^a^
	Control (n = 925)	Telemedicine (n = 257)	
**Clinical outcomes**			
Operating room delay, mean (SD) [95% CI], min^b^	7.8 (25.1) [5.1-10.5]	4.2 (11.1) [1.0-7.4]	.002
Procedure duration, mean (SD) [95% CI], min	134.4 (52.8) [130.9-137.8]	105.3 (41.5) [100.2-110.4]	<.001
Length of stay, mean (SD) [95% CI], d	1.9 (1.1) [1.8-1.9]	2.1 (1.0) [1.9-2.2]	<.001
Major adverse event			
Within 30 d^c^	35 (3.8) [3.0-5.7]	4 (1.6) [0.4-3.9]	.001
Death	1 (5.9)	0	NA
Reoperation	8 (47.1)	0	NA
Percutaneous or endoscopic intervention	4 (23.5)	3 (100)	NA
Pulmonary embolism and deep vein thrombosis	0	0	NA
Failure to be discharged within 30 d	0	0	NA
Other	4 (23.5)	0	NA
31-60 d^d^	20 (2.2) [1.3-3.3]	4 (1.6) [0.4-3.9]	<.001
Death	0	0	NA
Reoperation	2 (13.3)	0	NA
Percutaneous or endoscopic intervention	7 (46.6)	4 (100)	NA
Pulmonary embolism and deep vein thrombosis	0	0	NA
Other	6 (40.0)	0	NA
Hospital utilization			
ED visit within 30 d	174 (18.8) [16.3-21.4]	46 (17.9) [13.2-22.6]	.03
Readmission within 30 d	93 (10.1) [8.1-12.0]	17 (6.6) [3.9-10.4]	.02

Abbreviations: ED, emergency department; NA, not applicable.

^a^ *P* ≤ .05 signifies that the telemedicine group is not inferior to the control group. Unlike the conventional usage of *P *values to signify difference, here significant *P *values reject the null hypothesis to identify cases in which receiving telemedicine care was not significantly different than in the control group.

^b^ A total 326 patients included in the control group; 49 patients included in the telemedicine.

^c^ Events occurring within 30 days had data available for event type for 17 cases in the control group and 3 cases in the telemedicine group.

^d^ Events occurring between 31 and 60 days had data available for event type for 15 cases in the control group and 4 cases in the telemedicine group.

Hospital utilization in the telemedicine group was noninferior to the control group. We observed no differences in the frequency of ED visits (18.8%; 95% CI, 16.3%-21.4% vs 17.9%; 95% CI, 13.2%-22.6%; *P* = .03) or hospital readmission (10.1%; 95% CI, 8.1%-12.0% vs 6.6%; 95% CI, 3.9%-10.4%; *P* = .02) between the control and telemedicine groups (Table 2).

We conducted stepwise logistic regression modeling to estimate ER visits, hospital readmission, MAE within 30 days and MAE between 31 and 60 days. Age (odds ratio [OR], 0.98; 95% CI, 0.97-0.99; *P* = .01), type of bariatric surgery (SG vs RNY: OR, 0.69; 95% CI, 0.50-0.94; *P* = .02), and employment status (disabled vs employed: OR, 2.33; 95% CI, 1.10-4.92; *P* = .03) were associated with ER visits within 30 days (eTable 1 in Supplement 1). Type of bariatric surgery was associated with higher likelihood of hospital readmission, with sleeve gastrectomy associated with lower odds of both 30-day hospital readmission (OR, 0.44; 95% CI, 0.28-0.69; *P* < .001) and 30-day MAE (OR, 0.12; 95% CI, 0.04-0.40; *P* = .001) (eTable 2 and 3 in Supplement 1). There were no variables that were associated with higher likelihood of MAE between 31 and 60 days, and telemedicine use was not associated with any of the above outcomes (eTables 1-4 in Supplement 1).

## Discussion

To our knowledge, this study was one of the largest cohorts of patients undergoing bariatric surgery after total telemedicine preoperative care. We found that postoperative clinical outcomes and hospital utilization for patients undergoing bariatric surgery after exclusively telemedicine-based preoperative evaluation are noninferior to patients receiving traditional, in-person care. The advantages of telemedicine with regards to patient cost and time savings may facilitate accessibility to bariatric surgery, especially for the underserved.^19^

Bariatric surgery is the most effective treatment for medically refractory weight loss in people with severe obesity; however, low-income patients and patients from minoritized racial and ethnic groups have difficulty accessing health care, which makes them less likely to consider bariatric surgery as a treatment option.^20^ While bariatric surgery is safe, effective, and economical,^21,22,23^ it is considerably underutilized. Less than 1% of eligible patients with obesity undergo bariatric surgery,^24,25^ despite the widely encompassing referral criteria for adults laid forth by the National Institutes of Health.^26^ Since the late 1990s, the proportion of non-White individuals undergoing bariatric surgery has increased as well as those in the lowest income quartile.^27^ However, there remains a discordance between populations most affected by obesity and those obtaining surgery.

Obesity rates are highest in adults who are non-Hispanic Black, middle-aged, did not graduate from high school, and reside in the Midwest or Southern US.^28^ Prior studies have concluded that patients eligible for bariatric surgery based on BMI are more likely to be minority, have low socioeconomic status, be underinsured or uninsured, and have less access to health care.^29^ Yet, these eligible patients are not reflected proportionally in the demographics of those frequently being referred to or who undergo surgery.^30^ Individuals choosing surgery are largely women, White, urban-dwelling, have higher income, and have better access to health insurance.^20,31,32,33^ Overall, bariatric surgery is less accessible to patients with low income or from racial and ethnic minority groups, which exacerbates health inequity.

Telemedicine is revolutionizing the way health care is delivered, particularly during the 2020 SARS-CoV-2 pandemic. Prior to the COVID-19 public health crisis, telemedicine demonstrated clear advantages by improving access to care in geographically disadvantaged rural and global populations.^34,35,36,37,38^ Nevertheless, public health professionals were concerned that the use of telehealth could exacerbate existing health disparities with higher usage among younger, employed, urban-dwelling patients.^39,40,41^ Access to broadband internet and internet-capable devices is not equitably distributed. Individuals with lower household income and those with less educational achievement are more likely forego internet use; however, there is no difference in race or community type (ie, rural or urban).^42^ While telemedicine may theoretically expand access to high-quality health care and offer patients more flexibility, it is imperative that we remain mindful of how it may affect health equity.

Consistent with previous research, our study found that the telemedicine group was statistically younger than the in-person control group, but this difference is likely not clinically significant as the age difference was merely 3 years. Our study demonstrated no differences in race or ethnicity between telemedicine and traditional preoperative care groups. We did observe a higher frequency of unemployed patients in the telemedicine group, yet a higher percentage of these patients had private insurance. It is possible that patients able to seek bariatric surgery during the COVID-19 pandemic were more likely those with private insurance who were temporarily unemployed rather than essential workers, who are more likely to have government-sponsored insurance or be under- or uninsured.^43^ Subsequently, employment status and insurance coverage of the telemedicine group may reflect transient social changes resulting from the coronavirus pandemic. While most patients in both groups underwent laparoscopic RNY gastric bypass, a larger percentage of the telemedicine group had laparoscopic SG, reflecting the national trend toward more patients electing SG and fewer opting for RNY.^44^

Preoperative BMI was similar across the telemedicine and control groups. However, recent postoperative BMI was lower in the control group. This is potentially a result of an inherently shorter follow-up period, often less than 1 year postoperatively, in the telemedicine group due to study design. Additionally, recent BMI measurement was included in only those patients who had a BMI within 2 years of data collection. For the control group, 805 of 925 patients had an eligible recent BMI measurement. All patients in the telemedicine group had a recent BMI measurement. Patients in the control group with no recent BMI measurement (ie, lost to follow-up) were excluded from this analysis. As such, the difference noted between the 2 groups may be a result of loss to follow-up bias. The control cohort had a larger prevalence of any comorbid condition compared with the telemedicine group. Again, this may be a secondary effect from the pandemic, which disproportionately affected those with obesity and associated medical conditions.^45^ It is reasonable to assume that individuals with obesity and multiple comorbid conditions may have been less likely to seek medical care due to increased health consequence from exposure to COVID-19, especially for elective procedures such as bariatric surgery.

Overall, we observed no inferiority in the telemedicine group compared with the control group with regards to clinical outcomes and hospital utilization after bariatric surgery. There was no difference in surgical efficiency as measured by operating room delay and procedure duration. Patient clinical variables, including length of hospital stay and major adverse events up to 60 days postoperatively were also found to be noninferior between the 2 groups. Lastly, hospital utilization measured by postoperative ER visits and hospital readmission within 30 days were noninferior in the telemedicine group compared with the control group. After performing adjusted analyses using stepwise logistic regression, we found that telemedicine was not associated with a higher risk of 30-day ED visit, 30-day hospital readmission, or major adverse events.

### Limitations

This study had several limitations. It was a single institution and single surgeon study, which may limit external generalizability. The retrospective nature of the study may also introduce unintended bias. Additionally, all individuals in the telemedicine group had preoperative evaluation and bariatric surgery during the COVID-19 pandemic, which may have resulted in inherently discrepant cohort characteristics and possibly patient care. For the telemedicine group, documented patient weight during follow-up encounters was largely self-reported, which was at risk for inaccuracies due to recall and social desirability biases. Additionally, we were only able to collect ER visits from our institution, which may have missed patients who presented to outside emergency centers. Moreover, the postoperative follow-up period was limited to 60 days, which may have limited annotation of more long-term complications.

## Conclusions

In this cohort study, total preoperative telemedicine in bariatric surgery was associated with noninferior clinical outcomes and hospital utilization compared with traditional, in-person patient care. Telemedicine may expand the reach of bariatric surgery and narrow disparities for historically disinvested patient populations. Further investigations should focus on geographical differences between telemedicine and traditional, in-person patient populations and ensure both patient and clinician satisfaction. In addition, the total telemedicine design should be studied prospectively to identify patient and provider barriers to its use. Future implementation and dissemination may be beneficial in other surgical fields.

## Notes

Supplement 1. **eTable 1.** Logistic Regression Model Projecting 30-day ER Visit **eTable 2.** Logistic Regression Model Projecting 30-day Hospital Readmission **eTable 3.** Logistic Regression Model Projecting 30-day Major Adverse Events **eTable 4.** Logistic Regression Model Projecting 31-60 day Major Adverse Events

Supplement 2.
Data Sharing Statement
