# Supplementary material for: Clinical Outcomes and Hospital Utilization Among Patients Undergoing Bariatric Surgery With Telemedicine Preoperative Care
Source: JAMA Netw Open. 2023 Feb 10;6(2):e2255994. doi: 10.1001/jamanetworkopen.2022.55994 (PMC9918871; doi:10.1001/jamanetworkopen.2022.55994)
Supplement: Supplement 1. — eTable 1. Logistic Regression Model Projecting 30-day ER Visit eTable 2. Logistic Regression Model Projecting 30-day Hospital Readmission eTable 3. Logistic Regression Model Projecting 30-day Major Adverse Events eTable 4. Logistic Regression Model Projecting 31-60 day Major Adverse Events [file jamanetwopen-e2255994-s001.pdf]

Supplemental Online Content

Hlavin C, Ingraham P, Byrd T, et al. Clinical outcomes and hospital utilization among patients undergoing bariatric surgery with telemedicine preoperative care. *JAMA Netw Open.* 2023;6(2):e2255994. doi:10.1001/jamanetworkopen.2022.55994

eTable 1. Logistic Regression Model Projecting 30-day ER Visit

eTable 2. Logistic Regression Model Projecting 30-day Hospital Readmission

eTable 3. Logistic Regression Model Projecting 30-day Major Adverse Events

eTable 4. Logistic Regression Model Projecting 31-60 day Major Adverse Events

This supplemental material has been provided by the authors to give readers additional information about their work.

© 2023 Hlavin C et al. *JAMA Network Open.*

eTable 1. Logistic Regression Model Projecting 30-day ER Visit

Variable	Odds Ratio	95% CI	p-value
Telemedicine	0.96	0.66 – 1.38	0.81
Age	0.98	0.97 – 0.99	0.01
Sleeve gastrectomy (vs. RNY)	0.69	0.50 – 0.94	0.02
Unemployed (vs. Employed)	1.38	0.94 – 2.03	0.10
Retired (vs. Employed)	1.91	0.87 – 4.18	0.11
Disabled (vs. Employed)	2.33	1.10 – 4.92	0.03
Student (vs. Employed)	1.17	0.48 – 2.82	0.73

© 2023 Hlavin C et al. *JAMA Network Open.*

eTable 2. Logistic Regression Model Projecting 30-day Hospital Readmission

Variable	Odds Ratio	95% CI	p-value
Telemedicine	0.67	0.39 – 1.15	0.15
Sleeve gastrectomy (vs. RNY)	0.44	0.28 – 0.69	<0.001

© 2023 Hlavin C et al. *JAMA Network Open.*

eTable 3. Logistic Regression Model Projecting 30-day Major Adverse Events

Variable	Odds Ratio	95% CI	p-value
Telemedicine	0.45	0.16 – 1.28	0.13
Sleeve gastrectomy (vs. RNY)	0.12	0.04 – 0.40	0.001

© 2023 Hlavin C et al. *JAMA Network Open.*

eTable 4. Logistic Regression Model Projecting 31-60 day Major Adverse Events

Variable	Odds Ratio	95% CI	p-value
Telemedicine	0.71	[0.24 – 2.10]	0.54

© 2023 Hlavin C et al. *JAMA Network Open.*
