# Supplementary material for: Clinical Outcomes and Hospital Utilization Among Patients Undergoing Bariatric Surgery With Telemedicine Preoperative Care
Source: JAMA Netw Open. 2023 Feb 10;6(2):e2255994. doi: 10.1001/jamanetworkopen.2022.55994 (PMC9918871; doi:10.1001/jamanetworkopen.2022.55994)
Supplement: Supplement 2. — Data Sharing Statement [file jamanetwopen-e2255994-s002.pdf]

Data Sharing Statement

Hlavin. Clinical Outcomes and Hospital Utilization Among Patients Undergoing Bariatric Surgery With Telemedicine Preoperative Care. *JAMA Netw Open*. Published February 10, 2023. doi:10.1001/jamanetworkopen.2022.55994

Data
Data available: Yes
Data types: Deidentified participant data
How to access data: hlavinca@upmc.edu
When available: With publication

Supporting Documents
Document types: None

Additional Information
Who can access the data: Researchers requesting the data and whose proposed use of the data has been approved.
Types of analyses: For any purpose.
Mechanisms of data availability: After approval of the proposal and with a signed data access agreement.
